# Long-Term Outcomes of Guselkumab and Risankizumab for the Management of Moderate-to-Severe Psoriasis in the Elderly: Results from a Real-World Retrospective Study

**DOI:** 10.3390/jcm14186472

**Published:** 2025-09-14

**Authors:** Ioannis-Alexios Koumprentziotis, Irene Stefanaki, Eleni Routsi, Charitomeni Vavouli, Pantelis Panagakis, Marina Papoutsaki, Maria Politou, Aristeidis Vaiopoulos, Evdoxia Panou, Vasiliki Chasapi, Alexander Stratigos, Electra Nicolaidou

**Affiliations:** 1st Department of Dermatology and Venereology, “Andreas Sygros” Hospital for Skin Diseases, National & Kapodistrian University of Athens Medical School, 16121 Athens, Greece; irenestefanaki@hotmail.com (I.S.); routsie@gmail.com (E.R.); charitomenivavouli@hotmail.com (C.V.); panagakis98@yahoo.gr (P.P.); marinapapoutsaki@hotmail.com (M.P.); marypdoc@yahoo.gr (M.P.); avaiopoulos@gmail.com (A.V.); ev_panou@yahoo.gr (E.P.); chasapiresearch@gmail.com (V.C.); alstrat2@gmail.com (A.S.); electra.nicol@gmail.com (E.N.)

**Keywords:** psoriasis, biologics, elderly, guselkumab, risankizumab, interleukin-23

## Abstract

**Background/Objectives**: The treatment landscape of psoriasis has changed dramatically with the emergence of novel biological agents such as those targeting interleukin (IL)-23. Despite their high efficacy, evidence regarding their effectiveness and tolerability in elderly patients is currently limited. The number of older adults living with psoriasis is constantly increasing, highlighting the need for evidence-based guidance focused on this population. The aim of this study was to summarize our center’s experience with the IL-23 inhibitors guselkumab and risankizumab for the treatment of moderate-to-severe psoriasis in patients aged ≥65 years. **Methods**: We retrospectively reviewed all charts of moderate-to-severe psoriasis patients who received at least one dose of guselkumab or risankizumab (tildrakizumab is not available for use in Greece as of this date) and included those aged ≥65 years at the time of drug initiation. Disease severity was assessed using the Psoriasis Area and Severity Index (PASI), which was calculated at baseline and each subsequent visit up to 156 weeks of treatment. Treatment responses were evaluated with the percentages of patients achieving PASI75/90/100 (reductions from baseline PASI by 75, 90, and 100%, respectively) and absolute PASI ≤ 1 and PASI ≤ 3. All adverse events (AEs) were assessed and documented. The drug survival was estimated using the Kaplan–Meier estimate. **Results**: In total, 93 elderly patients (64 risankizumab and 29 guselkumab) were included. Regarding risankizumab’s effectiveness, PASI75 response rates increased from 77.4% at week 12 to 90.9% at week 52 and remained stable at 90.5% by week 156. Corresponding PASI100 responses were 67.7%, 81.8%, and 80.9%, respectively. Guselkumab-treated patients exhibited PASI75 response rates of 71.4% at week 12, which improved to 91.3% at week 52 and 100% for those evaluated at week 156, with PASI100 responses of 57.1%, 73.9%, and 83.3%. During observation, 6 (9.4%) risankizumab and 2 (6.9%) guselkumab patients discontinued medication. No statistically significant differences were observed regarding drug survival between patients aged ≥65 vs. <65 years. No serious AEs occurred, and no patient discontinued medication due to AEs. **Conclusions**: Both guselkumab and risankizumab demonstrated sustained efficacy and persistence along with a favorable safety profile in elderly patients with psoriasis over a three-year period. While our study is limited by its retrospective nature, our findings support the use of IL-23 inhibitors in this growing patient population.

## 1. Introduction

Psoriasis is a chronic, immune-mediated inflammatory skin disease, driven by a complex interplay of genetic, environmental, and immunological factors. Psoriasis affects approximately 2–4% of the global population. And while it can present at any age, incidence peaks are observed in early adulthood (15–25 years) and again between the ages of 50 and 60 [1,2,3,4,5]. Moreover, the global population is aging, and the prevalence of psoriasis in elderly patients—typically defined as those aged 65 years and above—is rising accordingly [6,7]. Epidemiological studies indicate that up to 15% of elderly patients with psoriasis have moderate-to-severe disease, necessitating systemic therapy. The clinical management of psoriasis in this population is complicated by age-related physiological changes, polypharmacy, and the presence of multiple comorbidities, all of which can impact treatment selection, safety, and outcomes [2,3,4,5,6,7]. Furthermore, elderly patients are frequently underrepresented in randomized controlled trials, resulting in a paucity of robust, long-term data to guide therapeutic decisions in this group [8,9,10,11,12].

Biological agents targeting interleukin (IL)-23 and IL-17 have revolutionized the management of moderate-to-severe plaque psoriasis, offering high efficacy and favorable safety profiles compared to traditional systemic agents. Among these, risankizumab and guselkumab—both selective IL-23p19 inhibitors—have emerged as leading options due to their durable efficacy, infrequent dosing schedules, and low rates of serious adverse events [9,13,14,15,16,17,18,19,20]. Post-marketing surveillance and real-world data have not revealed any new safety concerns specific to elderly patients; however, careful monitoring for infections and malignancies remains important due to the higher baseline risk in this population [6]. Despite this, the real-world performance of these agents in elderly patients, particularly with respect to long-term drug survival, safety, and tolerability, remains incompletely characterized [7,8,19].

Several large registry-based and multicenter cohort studies have demonstrated that drug survival of biologics is generally reduced in elderly patients compared to younger cohorts, with higher rates of discontinuation due to adverse events and, to a lesser extent, ineffectiveness [8,9,11,19]. For example, a multicenter European study of over 4000 patients found that elderly individuals (≥65 years) had a significantly increased risk of biologic discontinuation, particularly with IL-23 inhibitors, although within the elderly subgroup, IL-23 inhibitors (including risankizumab and guselkumab) exhibited higher drug survival than IL-17 inhibitors [15]. Similarly, registry data from the Czech Republic have shown that while the efficacy of biological treatment was comparable between older and younger patients, the drug survival rate among older patients was notably lower during the treatment period [19]. Notably, real-world studies consistently report that the long-term efficacy of IL-23 inhibitors in elderly patients is maintained, with PASI responses and quality-of-life improvements like those observed in younger populations [7,8,19,21,22,23,24].

While phase III trials and open-label extension studies have established the sustained efficacy and safety of risankizumab and guselkumab over several years, these studies have typically included few elderly participants [20,22,23].

Comorbidities and frailty are central considerations in the management of psoriasis in the elderly. This population frequently presents multiple coexisting conditions that exhibit high rates of hypertension, diabetes, obesity, coronary artery disease, and joint involvement, all of which may influence both the choice and tolerability of systemic therapies [1,2]. Frailty, defined by reduced physiological reserve and heightened vulnerability to stressors, adds complexity to therapeutic decisions and may predispose patients to complications. Polypharmacy and age-related declines in renal and hepatic function increase the risk of drug–drug interactions and adverse events, necessitating careful selection and monitoring of systemic agents [1,7,24,25]. Biologics, particularly IL-23 inhibitors, are generally favored in this context due to their targeted mechanism of action, minimal systemic toxicity, and low potential for pharmacokinetic interactions [7,9,26]. Treatment goals should be guided by a patient-centered approach, emphasizing quality of life, functional status, and minimizing adverse events and drug interactions [16,25].

Simplified dosing regimens and treatment convenience are key to maximizing adherence and reducing the burden of care in elderly patients with psoriasis. Prioritizing treatments with simple administration schedules, citing safety and ease of use as fundamental for older individuals with polypharmacy and physical limitations. Complex regimens—like frequent topical applications—often result in low long-term adherence, especially when patients lack caregiver support [25].

Altered pharmacokinetics and pharmacodynamics in the elderly further complicate systemic therapy. Age-related changes in body composition, decreased renal and hepatic clearance, and immunosenescence can affect drug absorption, distribution, metabolism, and elimination, potentially increasing susceptibility to adverse events [7,25]. However, the monoclonal antibody structure and subcutaneous administration of risankizumab and guselkumab confer predictable pharmacokinetics, with no requirement for dose adjustment based on age or renal/hepatic function [22,26,27].

The need for therapies with low rates of adverse events is paramount in the elderly, who are more vulnerable to infections, malignancy, and other complications of immunosuppression [19,20,21,22,23,24,25]. The accumulation of robust, long-term, real-world evidence is essential to optimize therapeutic strategies and improve outcomes for elderly patients with psoriasis. In Greece, tildrakizumab, another anti-IL-23 inhibitor, has not yet received marketing authorization and is therefore unavailable for clinical use. Consequently, this retrospective cohort study aimed to summarize our experience on the use of the two approved anti-IL-23 agents for psoriasis in Greece—risankizumab and guselkumab—over a follow-up period of up to 156 weeks.

## 2. Materials and Methods

This retrospective, single-center observational study included medical records of elderly (≥65 years) patients with moderate-to-severe plaque psoriasis treated with either guselkumab or risankizumab at the Psoriasis Outpatient Clinic of the 1st Department of Dermatology and Venereology, National and Kapodistrian University of Athens Medical School, ‘Andreas Sygros’ Hospital, in Athens, Greece. The study was approved by the hospital ethics committee, and all participants gave verbal informed consent for the use of their medical data. The study complied with the principles laid down in the Declaration of Helsinki.

Eligibility criteria included (i) a diagnosis of chronic (≥6 months) moderate-to-severe plaque psoriasis; (ii) age at treatment initiation ≥ 65 years; and (iii) the administration of at least 1 dosage of risankizumab or guselkumab. Guselkumab and risankizumab were administered at the standard dosing schemes and were initiated after a 3–4 week washout period when a previous biological agent or systemic treatment was used. Tildrakizumab was not included in the study, as this IL-23 inhibitor is not available for the treatment of in Greece.

Baseline demographic and clinical data were extracted from patient records, including gender, age at drug initiation, disease duration, body mass index (BMI), medical comorbidities, personal history of special locations affected (nails, scalp, genitals, palmoplantar area), concomitant psoriatic arthritis and number and type of previous treatments. To assess disease severity, the main measure utilized was the psoriasis area severity index (PASI), which was evaluated at baseline and during each sequential visit for each patient. The primary outcomes regarding treatment effectiveness at each follow-up visit (12, 24, 52, 104 and 156 weeks) included PASI 75/90/100 (reduction in baseline PASI score by 75/90/100%), PASI ≤ 3 and PASI ≤ 1 (as observed analysis). The Chi-squared and Fisher’s exact tests were used to determine the statistical significance of the differences in values obtained at the different time points of treatment for the categorical variables examined (PASI 75/90/100, PASI ≤ 3 and PASI ≤ 1). All adverse events (AEs) were physician-assessed and documented. To estimate treatment persistence, the drug survival was estimated using the Kaplan–Meier estimate. The log-rank test was utilized to compare the drug survival of elderly patients with that of all those <65 years old who have been treated and documented in our department’s database. The SPSS 29.0 version (IBM Corp., Chicago, IL, USA) software was used for all statistical analyses, and *p* < 0.05 was considered statistically significant. Categorical data were summarized as numbers and percentages, while continuous data were summarized as means and standard deviations (SDs) when appropriate.

## 3. Results

### 3.1. Guselkumab

In total, 29 elderly patients (mean age 71.1 years, range 65–86 years) received guselkumab for the management of moderate-to-severe plaque psoriasis (Table 1). The majority were male (65.5%), and the mean PASI score was 7.7 (SD 4.9) at the time of treatment initiation. The mean psoriasis disease duration was 15.3 (SD 12.4) years. Medical comorbid conditions were common, with 82.8% of patients suffering from at least one comorbidity and 41.4% presenting with three or more. Cardiovascular (58.6%) and metabolic disorders (69.0%) were the most frequently observed comorbidities, while psychiatric conditions were reported in 27.5%. Only 17.2% had concomitant psoriatic arthritis (PsA), and prior biologic exposure was relatively high, while 44.8% of the patients were biologic-naive, 72.4% of patients were previously treated with ≥2 systemic therapies and 27.6% with ≥2 biologics. A history of hard-to-treat locations such as nail (34.5%), scalp (51.7%), genital area (27.6%), and palmoplantar psoriasis (24.1%) was documented in the cohort.

Clinical responses to guselkumab were rapid and sustained throughout the study period (Table 2). At week 12, the PASI 75/90/100 responses were achieved by 71.4/60.7/57.1% of the evaluated patients, respectively. Absolute PASI ≤ 1 and PASI ≤ 3 responses were noted in 64.2% and 78.6% of patients. By week 24, clinical response improved, with PASI 75/90/100 rates reaching 92.3/80.8%/65.4%, respectively, and PASI ≤ 3 achieved in 92.3%. Durable efficacy was maintained by week 52 (PASI 75/90/100: 91.3/78.3/73.9%) and beyond, with maximal responses observed during week 104 (PASI 75/90/100: 93.8/93.8/87.5%) for those who remained on treatment. By week 156, PASI 100 and PASI ≤ 1 were maintained in 83.3% of patients, and all patients achieved the PASI ≤ 3 endpoint.

**Figure 1:**
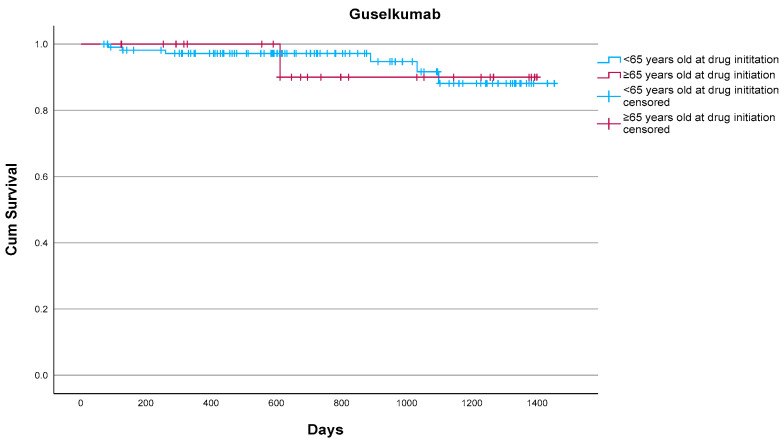
Drug survival of guselkumab in elderly (n = 29) and non-elderly patients (n = 111) as estimated by the Kaplan–Meier estimate. Overall, 2/29 (6.9%) elderly patients vs. 6/111 (5.4%) non-elderly patients discontinued guselkumab. No statistically significant differences were noted (*p* = 0.842) according to the log-rank test.

Guselkumab demonstrated excellent treatment persistence, with a 93.1% overall drug survival rate at the study endpoint. Only two (6.9%) of 29 patients discontinued treatment, both for reasons unrelated to serious adverse events. One patient had suffered from one episode of clinically mild stomatitis and, according to patient preference, changed to risankizumab with the adverse event improving after switching. The other patient discontinued due to secondary loss of efficacy. When compared with the non-elderly cohort treated at our center, drug survival did not differ in a statistically significant manner (*p* = 0.842) according to the log-rank test (Figure 1). Relatively low rates of adverse events were identified in the elderly group throughout the 3-year follow-up, with mild stomatitis being the only adverse event observed. Treatment was well tolerated, and no discontinuations were attributed to serious or life-threatening adverse events, serious infections, malignancies, or cardiovascular complications. These findings suggest that guselkumab demonstrates good effectiveness and tolerability in elderly populations with moderate-to-severe psoriasis, including those with multiple comorbidities, reinforcing its favorable long-term profile in this real-world setting.

### 3.2. Risankizumab

In total, 64 elderly patients (mean age 71.6 years, range 65–88 years) received risankizumab for moderate-to-severe plaque psoriasis (Table 1). The majority were male (59.4%), and the mean PASI score at treatment initiation was 7.4 (SD 5.5). The mean disease duration was longer compared to the guselkumab cohort at 21.6 (SD 15.2) years. Medical comorbidities were highly prevalent, with 85.9% of patients reporting at least one comorbidity and 40.6% presenting with three or more. Cardiovascular disorders were present in 62.5% of patients and metabolic disorders in 73.4%, while psychiatric conditions were documented in 10.9%. A substantial proportion (32.8%) had concomitant psoriatic arthritis, and prior exposure to systemic and biologic therapies was frequent. A total of 39.1% of patients were biologic-naïve, while 68.8% had previously received ≥2 systemic therapies and 34.4% had been exposed to ≥2 biologics. Involvement of hard-to-treat locations was common, with nail psoriasis in 57.8%, scalp psoriasis in 53.1%, palmoplantar psoriasis in 23.4%, and genital psoriasis in 21.9%.

Rapid and consistent responses to risankizumab were achieved and maintained across all timepoints (Table 2). At week 12, PASI 75/90/100 responses were achieved by 77.4/69.4/67.7% of the evaluated patients, respectively. Absolute PASI ≤ 1 and PASI ≤ 3 responses were observed in 70.9% and 83.9% of patients. At week 24, response rates demonstrated further improvement, with PASI 75/90/100 responses in 88.5/81.9/80.3%, respectively, and PASI ≤ 3 in 88.5% of patients. High levels of efficacy persisted during week 52 (PASI 75/90/100: 90.9/83.6/81.8%) and week 104 (PASI 75/90/100: 94.3/91.4/88.6%). At week 156, PASI 100 and PASI ≤ 1 responses were maintained in 80.9% of patients, and PASI ≤ 3 was achieved by 95.2%.

**Figure 2:**
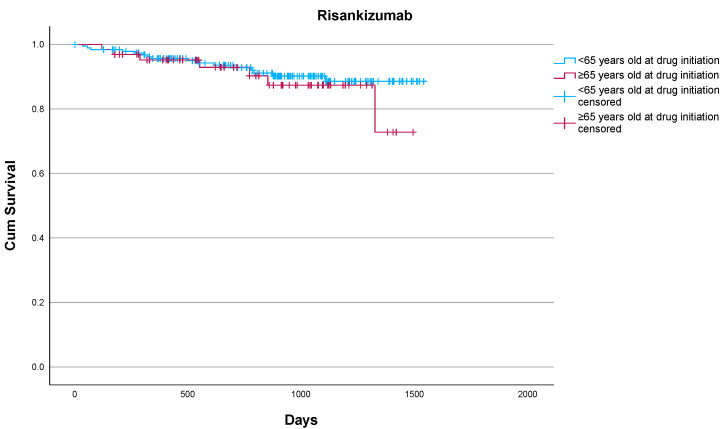
Drug survival of risankizumab in elderly and non-elderly patients as estimated by the Kaplan–Meier estimate.

Risankizumab also showed strong treatment persistence, with 89.1% of patients remaining on therapy during the study period. Discontinuation occurred in seven (10.9%) patients: two due to primary loss of efficacy, four due to secondary loss of efficacy (one due to insufficient psoriatic arthritis management, three due to skin disease), and one patient died from causes unrelated to treatment. No discontinuations were attributed to adverse events of any kind. Drug survival in elderly patients did not differ significantly from that of their younger counterparts treated at our center (*p* = 0.469) according to the log-rank test (Figure 2). Regarding safety, one patient reported blepharitis, and one patent reported muscle weakness and myalgias without discontinuing treatment. No serious adverse events emerged over the 3-year follow-up. Risankizumab was well tolerated, with no reported discontinuations due to serious infections, malignancies, cardiovascular events, or psychiatric complications. Our results underscore the favorable effectiveness and safety profile of risankizumab in elderly patients with moderate-to-severe plaque psoriasis, including those with a significant comorbidity burden and prior biologic exposure, supporting its suitability for safe long-term use in this patient population.

Overall, 7/64 (10.9%) elderly patients vs. 16/191 (8.4%) non-elderly patients discontinued risankizumab. No statistically significant differences were noted (*p* = 0.469) according to the log-rank test.

## 4. Discussion

The management of moderate-to-severe psoriasis in elderly patients remains challenging, primarily due to the coexistence of multiple comorbidities, age-related physiological changes, and the significant underrepresentation of this population in randomized clinical trials. In recent years, IL-23 inhibitors such as guselkumab and risankizumab have emerged as promising options, offering targeted efficacy with an improved safety profile, especially when compared with previously used systemic treatments. Our real-world experience provides valuable insight into the long-term effectiveness, tolerability and drug survival of these agents in an elderly cohort affected by significant comorbidities.

In both treatment groups, we observed sustained clinical responses that are consistent with or even exceed those reported in clinical trials and other real-world studies. PASI 90 and PASI 100 responses were achieved and maintained over the three-year follow-up, reflecting the durable efficacy of IL-23 inhibition in elderly patients, with no statistically significant differences observed between the two agents (Table 2). Despite this, the statistical analysis used to compare the two agents is limited by the relatively smaller number of guselkumab patients compared to those in the risankizumab cohort. Moreover, the high drug survival rates observed further support the sustained responses, with 93.1% remaining on guselkumab and 89.1% on risankizumab during the follow-up period.

In contrast to our findings, a large retrospective, multicentric, multi-country, cohort study demonstrated that elderly patients have an increased risk of biologic treatment discontinuation when compared to younger patients, particularly when being treated with IL-23 inhibitors [15]. When stratified analyses were conducted in elderly patients, those treated with IL-23 inhibitors had higher drug survival rates than those treated with IL-17 inhibitors [15]. Other studies have shown that effectiveness and safety results may be comparable across different age groups [5,6,28] and even in very elderly patients [29].

Apart from data focusing on efficacy and drug survival, previous studies with shorter follow-up periods have demonstrated the good safety profile of IL-23 inhibitors in elderly patients, with no new safety signals or higher rates of reported adverse events compared to younger patients [5,6,28]. This aligns with the available data from the large clinical trials that have led to the approval of those agents. Pooled analyses of long-term clinical trial data for risankizumab and guselkumab (up to 5 years) demonstrated that the rates of serious adverse events such as infections, malignancies, and major cardiovascular events remained low and stable over time, with no cumulative toxicity or increased risk in older adults [20,22,23,30].

Data arising from clinical trials should be interpreted cautiously, especially in populations that might not be adequately represented, thus not providing the necessary evidence for optimal patient management. A previously published systematic review, published back in 2019, has demonstrated that older adults might be poorly represented in RCTs studying systemic treatments in plaque psoriasis mainly because of a high rate of direct and indirect exclusion criteria [31]. This also highlights the need for long-term, real-world observations to gain more experience on the use of such agents in older patients. The inclusion of elderly patients and further subgroup analyses of clinical trials targeting older populations would provide meaningful data to support clinical decision-making.

Our study is mainly limited by its retrospective design and the relatively small sample size, particularly in the guselkumab cohort, which limits the generalizability of our findings. As a retrospective study, our data are inherently subject to selection bias and the absence of randomization. The sample size, while larger than in many previously published studies, remains modest and derived from a single center rather than a multicentric cohort. A significant strength of our study is its long-term follow-up, up to three years. It should be noted that long-term data beyond this time point remain limited, potentially hindering a comprehensive assessment of real-world adverse event rates in this frail population.

## 5. Conclusions

In conclusion, our findings contribute to the growing body of evidence supporting the use of IL-23 inhibitors in elderly patients with moderate-to-severe plaque psoriasis. Both guselkumab and risankizumab demonstrated high effectiveness when sustained for up to three years, along with a favorable safety profile. As the global population continues to age, life expectancy is increasing and psoriasis prevalence in the elderly keeps rising; as such, safe and durable treatment options are of the utmost importance for the long-term management of psoriasis in this population. Further research is warranted, including greater representation of elderly patients in randomized clinical trials, large multicenter prospective studies, and patient registries, to validate these findings and ultimately improve decision-making and the care of older patients.

## Figures and Tables

**Table 1:** Baseline characteristics of elderly patients treated with guselkumab and risankizumab.

Patient Baseline Characteristics	Risankizumab (n = 64)	Guselkumab (n = 29)
Female, n (%)	26 (40.6)	10 (34.5)
Age at drug initiation mean (range), years	71.6 (65–88)	71.1 (65–86)
Disease duration (yrs), mean (SD)	21.6 (15.2)	15.3 (12.4)
Baseline PASI, mean (SD)	7.4 (5.5)	7.7 (4.9)
BMI (kg/m^2^), mean (SD)	28.5 (5.3)	27.4 (5.8)
Comorbidities (yes), n (%)		
Cardiovascular disorders	40 (62.5)	17 (58.6)
Metabolic disorders	47 (73.4)	20 (68.9)
Psychological/psychiatric disorders	7 (10.9)	8 (27.6)
Specific comorbidities, n (%)		
Hypertension	37 (57.8)	15 (51.7)
Coronary artery disease	8 (12.5)	5 (17.2)
Diabetes mellitus	23 (35.9)	8 (27.6)
Dyslipidemia	38 (59.3)	16 (55.2)
Depression	4 (6.3)	5 (17.2)
Anxiety disorder	3 (4.7)	3 (10.3)
Multiple comorbidities ≥ 3, n (%)	26 (40.6)	12 (41.4)
Concomitant PsA, n (%)	21 (32.8)	5 (17.2)
Personal history of, n (%)		
Scalp psoriasis	34 (53.1)	15 (51.7)
Palmoplantar plaque psoriasis	15 (23.4)	7 (24.1)
Genital psoriasis	14 (21.9)	8 (27.6)
Nail psoriasis	37 (57.8)	10 (34.5)
Previous treatment with, n (%)		
Apremilast	35 (54.7)	16 (55.2)
Anti-TNFa	27 (42.2)	9 (31)
Interleukin-12/23 inhibitor	19 (29.7)	6 (20.7)
Interleukin-23 inhibitor	3 (4.7)	2 (6.9)
Interleukin-17 inhibitor	18 (28.1)	7 (24.1)
≥2 biologics	22 (34.4)	8 (27.6)
≥2 systemics	44 (68.8)	21 (72.4)
Biologic-naive	25 (39.1)	13 (44.8)

Abbreviations: n, number; BMI, body mass index; PsA, psoriatic arthritis; SD, standard deviation; yrs, years; PASI, psoriasis area severity index.

**Table 2:** Clinical outcomes of patients treated with guselkumab and risankizumab at each follow-up time point. The Chi-squared and Fisher’s exact tests were used to determine the statistical significance of the differences in values obtained (PASI 75/90/100, PASI ≤ 3 and PASI ≤ 1) at the different time points of treatment. *p*-values <  0.05 were considered statistically significant.

	Risankizumab	Guselkumab	*p*-Value
***Week 12***			
PASI 75	48/62 (77.4%)	20/28 (71.4%)	0.540
PASI 90	43/62 (69.4%)	17/28 (60.7%)	0.421
PASI 100	42/62 (67.7%)	16/28 (57.1%)	0.331
PASI ≤ 1	42/62 (70.9%)	18/28 (64.2%)	0.747
PASI ≤ 3	52/62 (83.9%)	22/28 (78.6%)	0.543
***Week 24***			
PASI 75	54/61 (88.5%)	24/26 (92.3%)	0.719
PASI 90	50/61 (81.9%)	21/26 (80.8%)	1.000
PASI 100	49/61 (80.3%)	17/26 (65.4%)	0.136
PASI ≤ 1	52/61 (85.2%)	21/26 (80.8%)	0.751
PASI ≤ 3	54/61 (88.5%)	24/26 (92.3%)	1.000
***Week 52***			
PASI 75	50/55 (90.9%)	21/23 (91.3%)	1.000
PASI 90	46/55 (83.6%)	18/23 (78.3%)	0.747
PASI 100	45/55 (81.8%)	17/23 (73.9%)	0.232
PASI ≤ 1	46/55 (83.6%)	18/23 (78.3%)	0.747
PASI ≤ 3	52/55 (94.5%)	22/23 (95.7%)	1.000
***Week 104***			
PASI 75	33/35 (94.3%)	15/16 (93.8%)	1.000
PASI 90	32/35 (91.4%)	15/16 (93.8%)	1.000
PASI 100	31/35 (88.6%)	14/16 (87.5%)	1.000
PASI ≤ 1	32/35 (91.4%)	15/16 (93.8%)	1.000
PASI ≤ 3	34/35 (97.1%)	15/16 (93.8%)	0.533
***Week 156***			
PASI 75	19/21 (90.5%)	12/12 (100%)	0.523
PASI 90	17/21 (80.9%)	10/12 (83.3%)	0.271
PASI 100	17/21 (80.9%)	10/12 (83.3%)	0.271
PASI ≤ 1	17/21 (80.9%)	10/12 (83.3%)	0.271
PASI ≤ 3	20/21 (95.2%)	12/12 (100%)	1.000

Abbreviations: PASI, psoriasis area severity index.

## Data Availability

The data supporting the findings in this study will be made available upon reasonable request.

## References

[1] Chen C., Che K., Guo Y., Huang Q., Hu X., Yu B. (2023). Effect of the age of onset on epidemiology, clinical features, and comorbidity of geriatric psoriasis. J. Dermatol..

[2] Kovitwanichkanont T., Chong A.H., Foley P. (2020). Beyond skin deep: Addressing comorbidities in psoriasis. Med. J. Aust..

[3] Armstrong A.W., Read C. (2020). Pathophysiology, clinical presentation, and treatment of psoriasis: A review. JAMA.

[4] Rosset F., Mastorino L., Dapavo P., Ortoncelli M., Quaglino P., Ribero S. (2023). Aging impact in response to different classes of biological treatment in psoriatic patients: A real-life observational study. J. Clin. Med..

[5] Hacınecipoğlu F., Çelik G., Kartal S.P. (2025). Efficacy and Safety of IL-17 and IL-23 Inhibitors in Elderly Patients with Plaque Psoriasis: A Real-World Study. J. Dermatol..

[6] Ruggiero A., Fabbrocini G., Cinelli E., Garza S.S.O., Camela E., Megna M. (2022). Anti-interleukin-23 for psoriasis in elderly patients: Guselkumab, risankizumab and tildrakizumab in real-world practice. Clin. Exp. Dermatol..

[7] Ter Haar E.L.M., Thomas S.E., van den Reek J.M.P.A., Otero M.E., Njoo M.D., Ossenkoppele P.M., de Jong E.M.G.J. (2022). Drug survival, safety, and effectiveness of biologics in older patients with psoriasis: A comparison with younger patients—A BioCAPTURE registry study. Drugs Aging.

[8] Ohata C., Anezaki H., Yanase T., Katayama E., Kaneko S., Saito K., Imafuku S. (2024). Real-world safety and efficacy of biologics in elderly patients with psoriasis: A multicenter observational study. J. Dermatol..

[9] Aval L.M., Yiu Z.Z., Alabas O.A., Griffiths C.E., Reynolds N.J., Hampton P.J., BADBIR Study Group (2025). Drug survival of IL-23 and IL-17 inhibitors versus other biologics for psoriasis: A British Association of Dermatologists Biologics and Immunomodulators Reister cohort study. J. Eur. Acad. Dermatol. Venereol..

[10] Zhu B., Jing M., Yu Q., Ge X., Yuan F., Shi L. (2022). Treatments in psoriasis: From standard pharmacotherapy to nanotechnology therapy. Adv. Dermatol. Allergol..

[11] Cline A., Cardwell L.A., Feldman S.R. (2017). Advances in treating psoriasis in the elderly with small molecule inhibitors. Expert Opin. Pharmacother..

[12] Van Winden M.E., van der Schoot L.S., Arias M.V.D.L.I., van Vugt L.J., van den Reek J.M., van de Kerkhof P.C., Lubeek S.F. (2020). Effectiveness and safety of systemic therapy for psoriasis in older adults: A systematic review. JAMA Dermatol..

[13] Yiu Z.Z., Becher G., Kirby B., Laws P., Reynolds N.J., Smith C.H., BADBIR Study Group (2022). Drug survival associated with effectiveness and safety of treatment with guselkumab, ixekizumab, secukinumab, ustekinumab, and adalimumab in patients with psoriasis. JAMA Dermatol..

[14] Torres T., Puig L., Vender R., Lynde C., Piaserico S., Carrascosa J.M., Chiricozzi A. (2021). Drug survival of IL-12/23, IL-17 and IL-23 inhibitors for psoriasis treatment: A retrospective multi-country, multicentric cohort study. Am. J. Clin. Dermatol..

[15] Chiricozzi A., Coscarella G., Puig L., Vender R., Yeung J., Carrascosa J.M., Torres T. (2024). Age affects drug survival rates of interleukin (IL)-17 and IL-23 inhibitors in patients with plaque psoriasis: Results from a retrospective, multicentric, multi-country, cohort study. J. Eur. Acad. Dermatol. Venereol..

[16] Napolitano M., Balato N., Ayala F., Patruno C., Patrì A., Megna M., Balato A. (2016). Psoriasis in elderly and non-elderly population: Clinical and molecular features. G. Ital. Dermatol. Venereol..

[17] Alabas O.A., Mason K.J., Yiu Z.Z., Smith C.H., Warren R.B., Griffiths C.E. (2025). Age and biologic survival in patients with moderate-to-severe psoriasis: A cohort study from the British Association of Dermatologists Biologics and Immunomodulators Register (BADBIR). Br. J. Dermatol..

[18] Ruggiero A., Fabbrocini G., Cinelli E., Megna M. (2022). Real world practice indirect comparison between guselkumab and risankizumab: Results from an Italian retrospective study. Dermatol. Ther..

[19] Sobotkova T., Hugo J., Salavec M., Kojanova M., Tichy M., Necas M., Rob F. (2025). Efficacy, safety, and drug survival during the first year of biologic therapy for psoriasis in elderly versus younger patients. Int. J. Dermatol..

[20] Papp K.A., Blauvelt A., Puig L., Ohtsuki M., Beissert S., Gooderham M., Lebwohl M.G. (2023). Long-term safety and efficacy of risankizumab for the treatment of moderate-to-severe plaque psoriasis: Interim analysis of the LIMMitless open-label extension trial up to 5 years of follow-up. J. Am. Acad. Dermatol..

[21] Gargiulo L., Ibba L., Malagoli P., Amoruso F., Argenziano G., Balato A., Narcisi A. (2024). Effectiveness, tolerability, and drug survival of risankizumab in a real-world setting: A three-year retrospective multicenter study—IL PSO (ITALIAN LANDSCAPE PSORIASIS). J. Clin. Med..

[22] Lebwohl M.G., Merola J.F., Rowland K., Miller M., Yang Y.W., Yu J., Langley R.G. (2023). Safety of guselkumab treatment for up to 5 years in patients with moderate-to-severe psoriasis: Pooled analyses across seven clinical trials with more than 8600 patient-years of exposure. Br. J. Dermatol..

[23] Blauvelt A., Tsai T.F., Langley R.G., Miller M., Shen Y.K., You Y., Puig L. (2022). Consistent safety profile with up to 5 years of continuous treatment with guselkumab: Pooled analyses from the phase 3 VOYAGE 1 and VOYAGE 2 trials of patients with moderate-to-severe psoriasis. J. Am. Acad. Dermatol..

[24] Galluzzo M., Marcelli L., Vellucci L., Paganini C., Maffei V., Tofani L., Talamonti M. (2023). Guselkumab for treatment of moderate-to-severe plaque psoriasis: Real-life effectiveness and drug-survival for up to 148 weeks. Expert Opin. Biol. Ther..

[25] Di Caprio R., Caiazzo G., Cacciapuoti S., Fabbrocini G., Scala E., Balato A. (2020). Safety concerns with current treatments for psoriasis in the elderly. Expert Opin. Drug Saf..

[26] Zhu Y., Xu Y., Zhuang Y., Piantone A., Shu C., Chen D., Sharma A. (2020). Evaluating potential disease-mediated protein-drug interactions in patients with moderate-to-severe plaque psoriasis receiving subcutaneous guselkumab. Clin. Transl. Sci..

[27] Suleiman A.A., Khatri A., Minocha M., Othman A.A. (2019). Population pharmacokinetics of the interleukin-23 inhibitor risankizumab in subjects with psoriasis and Crohn’s disease: Analyses of phase I and II trials. Clin. Pharmacokinet..

[28] Ibba L., Gargiulo L., Vignoli C.A., Alfano A., Cortese A., Valenti M. (2023). Effectiveness and safety of anti-IL-23 and anti-IL-17 biological therapies for psoriasis in elderly patients: Real-world experience from two Italian hospitals. J. Eur. Acad. Dermatol. Venereol..

[29] Lorenzoni E., DI Cesare A., Rosi E., Trovato E., Pescitelli L., Panduri S., Ricceri F., Rossari S., Magnano M., Savarese I. (2024). Risankizumab in very elderly patients in real-world practice. Ital. J. Dermatol. Venereol..

[30] Gordon K.B., Lebwohl M., Papp K.A., Bachelez H., Wu J.J., Langley R.G., Reich K. (2022). Long-term safety of risankizumab from clinical trials in patients with moderate-to-severe plaque psoriasis. Br. J. Dermatol..

[31] Schaap M.J., Van Winden M.E., Seyger M.M., de Jong E.M., Lubeek S.F. (2020). Representation of older adults in randomized controlled trials on systemic treatment in plaque psoriasis: A systematic review. J. Am. Acad. Dermatol..

